# The response of wetland quality indicators to human disturbance indicators across the United States

**DOI:** 10.1007/s10661-019-7323-5

**Published:** 2019-06-20

**Authors:** Alan T. Herlihy, Jean C. Sifneos, Gregg A. Lomnicky, Amanda M. Nahlik, Mary E. Kentula, Teresa K. Magee, Marc H. Weber, Anett S. Trebitz

**Affiliations:** 1 Department of Fisheries & Wildlife, Oregon State University, 104 Nash Hall, Corvallis, OR 97331 USA; 2 Department of Statistics, Oregon State University, Corvallis, OR 97331 USA; 3 CSS Dynamac Corp, 200 SW 35th St., Corvallis, OR 97333 USA; 4 Department of Biology, Kenyon College, 202 N. College Road, Gambier, OH 43022 USA; 5 Present Address: National Health and Environmental Effects Research Laboratory-Western Ecology Division, US Environmental Protection Agency, 200 SW 35th St., Corvallis, OR 97333 USA; 6 National Health and Environmental Effects Research Laboratory- Mid-Continent Ecology Division, US Environmental Protection Agency, 6201 Congdon Blvd, Duluth, MN 55804 USA

**Keywords:** Wetlands, Lead, Phosphorus, Nitrogen, Vegetation, Human disturbance

## Abstract

We analyzed data from 1138 wetland sites across the conterminous United States (US) as part of the 2011 National Wetland Condition Assessment (NWCA) to investigate the response of indicators of wetland quality to indicators of human disturbance at regional and continental scales. The strength and nature of these relationships in wetlands have rarely been examined over large regions, due to the paucity of large-scale datasets. Wetland response indicators were a multimetric index of vegetation condition (VMMI), percent relative cover of alien plant species, soil lead and phosphorus, and water column total nitrogen and total phosphorus. Site-level disturbance indices were generated from field observations of disturbance types within a circular 140-m radius area around the sample point. Summary indices were calculated representing disturbances for ditching, damming, filling/erosion, hardening, vegetation replacement, and vegetation removal. Landscape-level disturbance associated with agricultural and urban land cover, roads, and human population were based on GIS data layers quantified in 200, 500, and 1000-m circular buffers around each sample point. Among these three buffer sizes, the landscape disturbance indicators were highly correlated and had similar relationships with the response indictors. Consequently, only the 1000-m buffer data were used for subsequent analyses. Disturbance-response models built using only landscape- or only site-level disturbance variables generally explained a small portion of the variance in the response variables (*R*^2^ < 0.2), whereas models using both types of disturbance data were better at predicting wetland responses. The VMMI was the response variable with the strongest relationship to the disturbances assessed in the NWCA (national model *R*^2^ = 0.251). National multiple regression models for the soil and water chemistry and percent alien cover responses to disturbance indices were not significant. The generally low percentage of significant models and the wide variation in predictor variables suggests that stressor-response relationships vary considerably across the diversity of wetland types and landscape settings found across the conterminous US. Logistic regression modeling was more informative, resulting in significant national and regional models predicting site presence/absence of alien species and/or the concentration of lead in wetland soils above background.

## Introduction

There is an increasing demand for information that can enhance understanding of the ecological quality of the world’s wetland resources beyond status and trends in wetland extent or qualitative indicators of wetland function (e.g., Fennessy et al. 2007; Wardrop et al. 2013). Data on the ecological condition of wetlands can be used to report on the ambient status of the resource, target restoration and protection efforts; evaluate the effects of mitigation and restoration practices; support regulatory decisions; and track the impact of land-use decisions (Scozzafava 2009; Scozzafava et al. 2011; Wardrop et al. 2007a; Whigham et al. 2007). Accordingly, recent years have seen attention given to development of quantitative, field-based methods in support of wetland management and protection. These efforts have resulted in progress on development of new assessment methods, definition of reference condition, and design of protocols for obtaining a representative sample of wetlands (e.g., Fennessy et al. 2007; Stevens Jr. and Jensen 2007; Wardrop et al. 2007b; Whigham et al. 2007).

Wetlands are affected by a wide variety of human disturbances (hereafter, disturbance). Disturbance effects on wetlands vary greatly depending on wetland type, magnitude of the stress, and landscape setting. A disturbance may also have a significant effect at one scale, but be insignificant at larger or smaller scales. Many studies have illustrated the effects of different land uses and other disturbances on wetland condition on a local or basin scale (e.g., Mensing et al. 1998; Houlahan and Findlay 2004; Hychka et al. 2007). The expectation that such relationships exist has formed the basis for the design of many water quality monitoring efforts (e.g., Puckett 1995). However, the strength and nature of these relationships in wetlands have rarely been examined over large regions, due to the paucity of large-scale datasets.

In 2011, the US Environmental Protection Agency (USEPA) conducted the National Wetland Condition Assessment (NWCA) across the conterminous US, sampling 1138 wetland sites to characterize vegetation, soil chemistry, water chemistry, and presence of anthropogenic disturbances. Landscape disturbances in 200-, 500-, and 1000-m circular buffers around each selected sample point were also quantified using available GIS data layers. Our objectives were to use the large-scale data to examine relationships between wetland response and disturbance. We examined these relationships using the GIS landscape data and site-level observations of disturbance, to determine (1) strength of these wetland response-disturbance relationships nationally and at smaller scales, (2) the relative effect of landscape versus local site–level disturbances, and (3) the wetland response indicators that were most related to disturbance. Analysis of the NWCA data provides a unique opportunity to investigate disturbance-response relationships in wetlands at large continental and regional scales using data designed and collected for this purpose.

## Methods

### NWCA overview and study variables

The purpose of the USEPA’s National Aquatic Resource Surveys (NARS) is to generate statistically valid and environmentally relevant reports on the condition of the nation’s aquatic resources every five years. The NWCA is one component of the NARS along with national surveys of lakes, streams, rivers, and near-coastal systems. The NWCA was designed to assess the regional ecological condition of broad groups or subpopulations of wetlands, rather than for individual wetlands or smaller spatial scales (e.g., individual states). The target population for the NWCA was all wetlands of the conterminous United States, not currently in crop production, including tidal and nontidal wetted areas with rooted vegetation and, when present, shallow open water less than 1 m in depth (Olsen et al. 2019). A wetland’s jurisdictional status under state or federal regulatory programs did not factor into this definition.

Details of the NWCA sampling design and site selection are described in the NWCA technical report (USEPA 2016a) and Olsen et al. (2019), and are briefly described here. Site selection was completed in two steps. A consistent national digital map of all wetlands in the conterminous US was not available; however, the US Fish and Wildlife Service (USFWS) conducts the National Wetland Status and Trends (S&T) survey periodically to assess wetland extent. The approximately 5000 4-mi^2^ plots from S&T were used to identify wetlands in the first step of site selection. In the second step, a Generalized Random Tessellation Stratified (GRTS) survey design (Stevens Jr. and Olsen 1999; Stevens Jr. and Olsen 2004) for an area resource was applied to the S&T wetland polygons and stratified by state with unequal probability of selection by NWCA wetland type (Olsen et al. 2019).

Sites from the NWCA survey design were screened using recent aerial photo interpretation and GIS analysis to eliminate locations not suitable for NWCA sampling (e.g., non-NWCA wetland types, wetlands converted to non-wetland land cover due to development). Sites might also be eliminated during field reconnaissance if, for example, they were a non-target type or could not be assessed due to accessibility or safety issues. Dropped sites were systematically replaced from a pool of replacement sites from the random design.

**Fig. 1:**
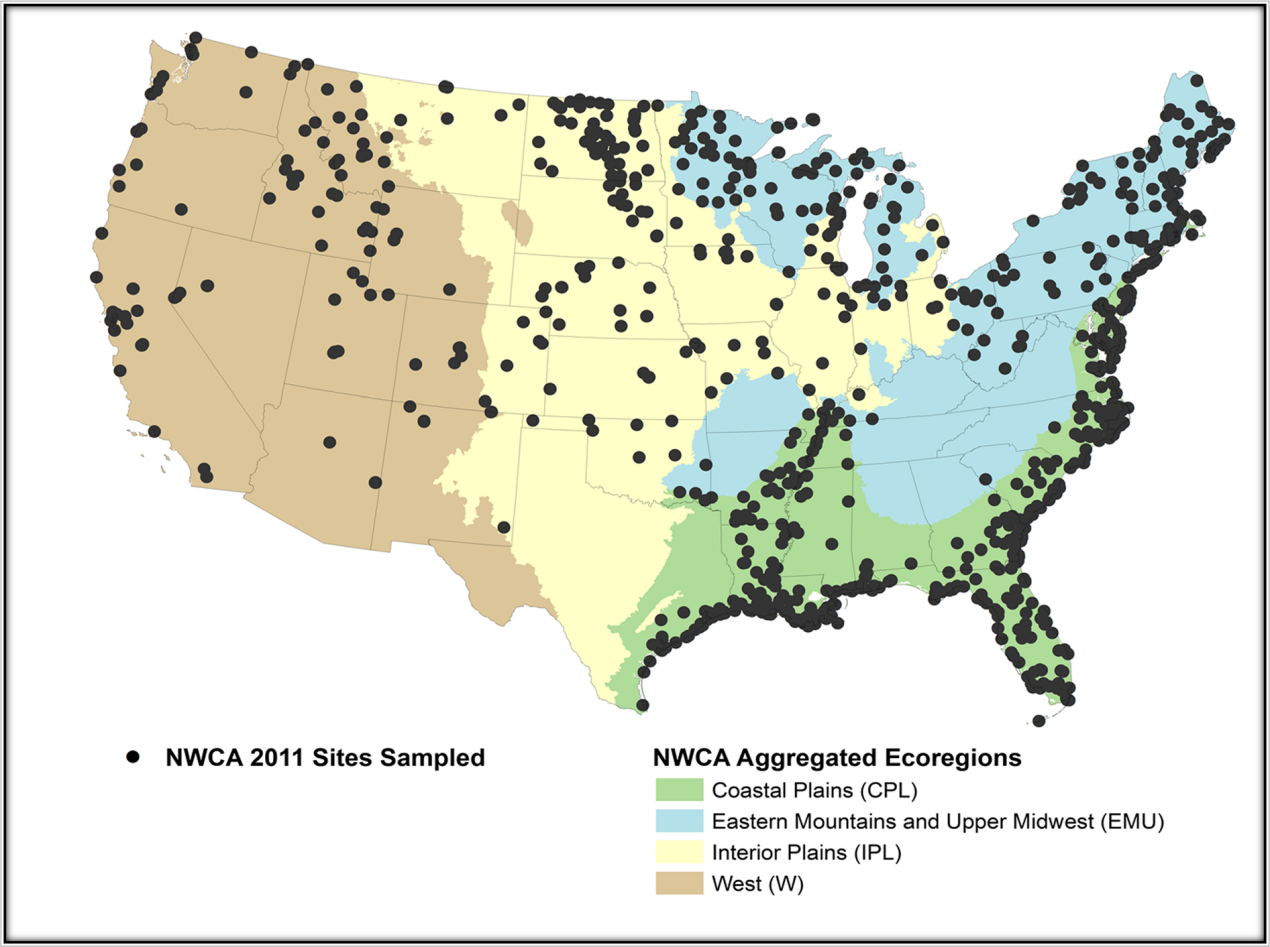
Location of sites sampled in the National Wetland Condition Assessment (NWCA) and the boundaries of the aggregated ecoregions used by the NWCA in the United States

A total of 1138 sites were sampled in the NWCA (Table 1), of which 967 were randomly selected probability sites used to make the national condition estimates in the NWCA report (USEPA 2016b). An additional 21 sites were probability sites from state intensification surveys that did not meet the design selection criteria for NWCA. The remaining 150 sites were handpicked in an effort to find least-disturbed reference sites (see Herlihy et al. 2019). As the objective of this paper was to analyze stressor-response relationships and not to make unbiased population estimates of wetland condition, all 1138 sites, both random and handpicked, were used in our analyses to maximize sample size. Sample sites were distributed throughout the conterminous US (Fig. 1). The spatial distribution across the country was not uniform, but paralleled the distribution of wetlands in the nation as represented in the S&T sample frame.

**Table 1:** Sample sizes in the National Wetland Condition Assessment (NWCA) and analyzed subpopulations

Full name	Code	Number of sites	Number without water data	Number without soil data
National	ALL	1138	522	99
NWCA aggregated ecoregion				
Coastal Plain	CPL	567	297	47
Eastern Mountains and Upper Midwest	EMU	214	92	3
Interior Plains	IPL	190	74	46
West	W	167	59	3
NWCA aggregated wetland type				
Estuarine herbaceous	EH	272	87	36
Estuarine woody	EW	73	38	2
Palustrine, riverine, or lacustrine-herbaceous	PRLH	358	131	52
Palustrine, riverine, or lacustrine-woody	PRLW	435	266	9
HGM class				
Depressions	Depressions	283	115	43
Flats	Flats	186	132	6
Riverine	Riverine	269	139	6

For our analyses, we studied the responses of a series of wetland ecological indicators against a variety of site-level field and GIS-derived landscape-level data describing disturbance variables (Table 2). We chose six ecological indicators as response variables: two biological measures, two soil chemistry indicators, and two water chemistry indicators. These particular variables are of high interest for ecological monitoring because they are responsive to disturbance and capture a wide variety of wetland ecological attributes (Trebitz et al. 2007; Stapanian et al. 2013). Site-level disturbance was described using the six variables in Table 2. The site-level variables were aggregate indices used in the 2011 NWCA reports to estimate stressor extent and relative risk (USEPA 2016a, 2016b). These six indices consolidated all of the disturbances observed during field sampling into a manageable number of site-level disturbance groupings for statistical analysis. We also selected seven GIS landscape-level variables for analysis (Table 2). These seven were selected because they have previously been associated with impact to surface waters and were readily available from national GIS data layers (Herlihy et al. 1998; Rooney and Bayley 2011).

**Table 2:** Ecological indicator (response) and disturbance (predictor) variables used in the analysis of the National Wetland Condition Assessment data

Variable	Code used in Figures and Tables
Ecological indicator variables (Response)	
Relative percent cover alien plants	%Alien
Vegetation multimetric index of condition	VMMI
Lead (mg/kg) in surface soil	Soil Pb
Total phosphorus (mg/kg) in surface soil	Soil P
Total nitrogen (μg/L) in water column	TN
Total phosphorus (μg/L) in water column	TP
Site-level disturbance variables (Predictor)	
Damming disturbance index	Dam
Ditching disturbance index	Ditch
Filling/Erosion disturbance index	Fill
Hardening disturbance index	Harden
Vegetation Removal disturbance index	VegRemoval
Vegetation Replacement disturbance index	VegReplace
Landscape-level disturbance variables (Predictor)	
Percent agriculture land use/land cover (LULC)	%Agr
Percent developed LULC	%Dev
Percent impervious surface	%Imp
Percent recreational LULC	%Rec
Human population density (number/mi^2^)	PopDen
Road density in buffer (km/km^2^)	RoadDen
Hydrologically modified (canal/ditch) length (km)	HydroMod

### Field and laboratory methods

Field and laboratory methods for the NWCA are described in detail by USEPA (2011a, b). Wetland sites were sampled in 2011 during an index period ranging from April to September depending on the growing season of the state in which the site was located. Sample collection focused on a 0.5-ha assessment area (AA) defined around each selected sample point. The AA was generally circular with a 40-m radius, but for very small or narrow wetlands, the AA shape was adjusted to a polygon or irregular shape to fit within the constraints of wetland boundaries. Within the AA, field crews (1) collected a water sample if standing water of sufficient depth (> 15 cm) to sink a pole-mounted dipper was present (Trebitz et al. 2019), (2) sampled soil (Nahlik et al. 2019) and vegetation (Magee et al. 2019a), and (3) completed checklists for presence of hydrologic alterations and other human disturbances (Lomnicky et al. 2019).

We focused our water chemistry analysis on total nitrogen (TN) and total phosphorus (TP) concentrations, which were measured in the laboratory by acid persulfate digestion and colorimetry. About half of the sites had no standing water, so they lacked water chemistry data (Table 1). See Trebitz et al. (2019) for more detail on water chemistry methods and additional results.

Four soil pit locations were systematically located in the AA and excavated to a depth of 60 cm. One soil pit was selected as representative of soil in the AA and expanded to a depth of 120 cm. At the representative pit, soil samples were collected for each soil layer more than 8 cm thick and sent to the lab for extensive chemical analysis (USEPA 2011a, 2011b). About 10% of the sites had no soil data due to difficulties in obtaining samples (Table 1). For this study, we focused only on lead and total soil phosphorus (soil P) concentration data from the uppermost layer collected and analyzed from each site. Almost all (97%) of sites from which soils were collected had chemistry data from a layer that began within 10 cm of the surface. In the laboratory, lead was measured by inductively coupled plasma mass spectroscopy (ICP-MS) and total phosphorus by a trace element procedure, which calls for a nitric and hydrochloric acid extraction and measurement by ICP-MS (USEPA 2011b).

Vegetation sampling methods are described in detail elsewhere (USEPA 2011a; Magee et al. 2019a) and summarized here. Five 100-m^2^ vegetation plots were systematically placed in the AA according to predetermined rules based on the shape of the AA. All vascular plants in each plot were identified to the lowest taxonomic level possible, typically to species. Taxa not readily identified in the field were collected and identified in the lab by regionally expert botanists. Percent cover for each species was estimated as a direct percentage (0–100%) of the 100-m^2^ area of each vegetation plot. Species trait information, including state-level coefficients of conservatism (*C*-values) and state-level native status, was gathered from literature or database sources, or in some cases developed, for each taxon-state pair observed in the NWCA (USEPA 2016a; Magee et al. 2019b).

The field data and species trait information were used to calculate numerous candidate metrics of vegetation condition, which were screened based on range, redundancy, repeatability, and responsiveness, for potential inclusion in a vegetation multimetric index (VMMI) that would serve as the principle indicator of biological condition for the NWCA (USEPA 2016b). VMMI development, calculation, and use are detailed in Magee et al. (2019a). Thousands of candidate VMMIs were evaluated using a series of objective performance criteria. The final VMMI was based on four broadly applicable component metrics: floristic quality assessment index, relative importance of native plants, number of plant species tolerant to disturbance, and relative cover of native monocots. This VMMI was scored to range from 0 to 100 with higher values reflecting better condition, and was applied nationally. However, to account for natural variation in the VMMI across the conterminous US, different VMMI value thresholds for delineating good, fair, and poor condition were defined for each of 10 ecoregion-by-wetland type groups. These condition thresholds were based on the distribution of VMMI values observed in least-disturbed sites for each ecoregion-by-wetland type group. In addition to the VMMI, we calculated a metric describing the relative percent cover of alien (introduced and adventive species) plants at each sample location (see USEPA 2016a for calculation). Hereafter, we refer to relative percent cover of alien plants as percent alien.

A checklist of hydrologic alteration observed in each AA was completed. In addition, a human disturbance checklist was completed at 13 10 × 10-m plots, one located at the AA center and 12 arranged in the study buffer surrounding the AA (Lomnicky et al. 2019). The 12 plots in the buffer were laid out in the four cardinal directions (3 in each direction): the first plot at the edge of the assessment area (40 m from the AA center), the second plot at the farthest extent of the study buffer (usually 140 m from the AA center), and the third plot midway between the other two. The human disturbance checklist data and the hydrologic alteration checklist data were categorized into six indicators of human disturbance: ditching, damming, filling/erosion, hardening, vegetation removal, and vegetation replacement (Table 2). A disturbance index was calculated for each category of site-level disturbance based on the proximity-weighted average of the number of human disturbances observed in each plot as described in Lomnicky et al. (2019). An index value of 0.59 would indicate that one human disturbance from the disturbance checklist was observed in each of the 13 plots at the site. The maximum value observed at a site in the NWCA for any of the six site-level disturbance categories was 2.2, but it was very rare for a site to have values greater than 1.

### GIS landscape data

We used available GIS data layers to identify landscape disturbance indicators for agriculture, development, impervious surface, recreation, road density, human population density, and hydrologic modification (Table 2). Disturbances from agriculture, development, impervious surface, road density, and population density were calculated for three circular buffers with 200-, 500-, and 1000-m radii around the randomly selected or handpicked sample point using ArcGIS software. Disturbances from recreation and hydrologic modification were only calculated for a circular buffer of 1000-m radius around the sample point. Agriculture, development, and impervious surface data layers were based on the 2006 National Land Cover Database (NLCD, Homer et al. 2007; Yang et al. 2003). Development included all four NLCD developed land classes (open space, low, medium, and high density) and agriculture included both pasture/hay and cultivated crop classes. Population density and road density data layers were obtained from 2010 US Census TIGER shapefiles (US Census Bureau 2010) and recreation disturbance was based on the USGS Protected Areas Database of the United States (PADUS; USGS 2012). We considered areas coded as national parks, trails, and landmarks, as well as recreation management areas, historic/cultural areas, state parks, and local recreation areas to be “Recreational.” Hydrologic modification was defined as the total length of canals or ditches in the buffer as represented in the National Hydrography Dataset Plus (NHDPlus) version 2 (McKay et al. 2012).

### Statistical analysis

About 8% of the sites were visited twice during the sampling index period. Only data from the first site visit was used in this paper; sample sizes are given in Table 1. Skewed response variables (soil lead, soil P, TN, and TP) were log transformed. Percent alien cover and soil lead were also investigated as binary variables. For binary analysis, percent alien cover was transformed to presence/absence of alien species and soil lead was transformed to levels above and below 35 mg/kg, which was the background level for lead used as a threshold to indicate human disturbance in the NWCA (Nahlik et al. 2019). We transformed all the disturbance variables to achieve a roughly common 0–10 data range to make comparisons more meaningful and to aid in the interpretation of the logistic regression odds ratios. Thus, all field-based disturbance indices were multiplied by 10, landscape percentage variables divided by 10, population density was log10 transformed, and hydrologically modified length was analyzed in kilometers.

We analyzed the NWCA data nationally and within different subpopulations (Table 1). Eleven subpopulations were defined based on NWCA ecoregions, NWCA aggregated wetland types, and hydrogeomorphic (HGM) classes (Table 1). Four aggregated ecoregions (Coastal Plain (CPL), Eastern Mountains and Upper Midwest (EMU), Interior Plains (IPL), and West (W), mapped in Fig. 1) were used for NWCA analysis and reporting (Herlihy et al. 2019) and each one represents a subpopulation for our analysis. The seven broad wetland types from the NWCA design were combined into four NWCA aggregated wetland types based on estuarine versus inland (palustrine, riverine, or lacustrine (PRL)) status and dominant vegetation (woody versus herbaceous) (Herlihy et al. 2019). Thus, the four vegetation subpopulations we considered were represented by the NWCA aggregated wetland types, estuarine-herbaceous (EH), estuarine-woody (EW), PRL-herbaceous (PRLH), and PRL-woody (PRLW). Shrub-scrub and forested types were considered woody, whereas, emergent, unconsolidated bottom, or aquatic bed types, and previously farmed emergent types were considered herbaceous. We evaluated three HGM (Brinson 1993) classes (depressions, flats, and riverine). HGM subpopulations with fewer than 50 samples (fringe and slope) were not analyzed. In addition, we did not analyze the tidal HGM class because it had virtually the same membership as the estuarine wetland types.

We conducted a series of exploratory analyses to better understand the behavior of predictor (disturbance) and response (ecological indicator) variables. First, we calculated a series of Pearson correlations to examine relationships of the GIS buffer widths (200, 500, and 1000 m), among the three land-cover/land-use disturbance variables (percent agriculture, developed land, and impervious surface), and also, between percent agriculture and the VMMI, nationally and for the 11 wetland subpopulations. Next, we looked at patterns in the distribution of value ranges for the 13 predictor variables and for the six response variables using box and whisker plots. Finally, we examined Pearson correlations among the 13 predictor variables and among the six response variables, nationally and by subpopulation.

Our next step was to perform 144 multiple regressions and 24 logistic regressions to evaluate relationships between all predictor and response variables. For the multiple regression analysis, this entailed looking at combinations of six responses and two disturbance predictor groups (site-level and GIS landscape) for the nation and 11 subpopulations (i.e., 12 total subpopulations). For the logistic regression analysis, the relationship between the two binary responses (soil lead above/below background concentration, alien species present/absent) and the disturbance variables was also investigated for each of the 12 subpopulations. Recognizing that we were doing multiple analyses on the same dataset, we chose a correction of 0.05 divided by the number of possible models (144 + 24 = 168). This resulted in a pseudo-significance value of 0.0003 that we used to identify meaningful models. This cutoff is loosely based on a Bonferroni type of adjustment for multiple comparisons where it is used to guard against false significance (Ramsey and Schafer 2013). We also used an additional cutoff of an adjusted *R*^2^ of 0.2 or greater to identify meaningful models. While lower *R*^2^ values may or may not be statistically significant, we felt that they explained too small a proportion of the variance in the response variables to be ecologically significant.

To investigate which set of disturbance variables, field or GIS landscape, predict the responses better, we looked at multiple regressions of the six response variables versus the field disturbance variables and versus the GIS landscape disturbance variables for the entire dataset and by subpopulation. For the regressions, the response/disturbance/subpopulation combinations that had models with adjusted *R*^2^ ≥ 0.2 and *p* values ≤ 0.0003 were further investigated and compared using an extra sum of squares *F*-test to see which of the two disturbance predictor sets, field or GIS landscape, explained more of the variation in the response. The extra sum of squares *F*-test determines the reduction in the sums of squares between a full and reduced model. The full model had all the field and GIS landscape variables in it and the reduced models had either just the landscape variables or just the field variables. A small difference in sums of squares between the full and reduced models would produce a small *F*-value and a large *p* value suggesting insufficient evidence to show that the models (full and reduced) were different in predicting the response.

We also sought to investigate the relative strengths of site-level disturbance variables versus GIS landscape disturbance variables. Multiple regression models with adjusted *R*^2^ ≥ 0.10 were evaluated to identify the response/subpopulation combinations that merited further investigation. For this set of response/subpopulation combinations, we then combined the site-level and GIS landscape predictor sets to make an overall disturbance model and conducted an exhaustive search to choose regression models based on the lowest Bayesian Information Criteria (BIC) value (Ramsey and Schafer 2013). Analyses were done using the LEAPS package in R (Lumley and Miller 2009). Only the combined site-level/landscape models with an adjusted *R*^2^ ≥ 0.2 and a *p* value ≤ 0.0003 were considered significant and were retained for reporting.

For the presence/absence variables, we regressed the binary responses for lead in wetland soils and alien species against the combined site-level and GIS landscape disturbance variables, did an exhaustive model search, and chose the model with the lowest BIC value using the BESTGLM package in R (McLeod and Xu 2014). We only reported models that had a *p* value ≤ 0.0003 as determined by a likelihood ratio test comparing the BIC model to the null model. The odds ratios associated with the predictors in these models were tallied and McFadden’s *R*^2^, which can be used as a goodness of fit statistic, was calculated.

## Results

### Effects of GIS buffer width

We investigated whether there were important relationships for disturbance variables associated with different GIS buffer radius lengths. The land-use/land-cover (LULC) data gathered from the three buffer widths were highly related to one another based on Pearson correlations (Table 3). Buffer LULC percentages for agriculture, developed land, and impervious surface were all correlated at *r* ≥ 0.7 across buffer sizes (Table 3). Correlations for each of these three variables between the 500- and 1000-m buffer sizes were all *r* ≥ 0.93, between the 200- and 500-m buffer widths were all *r* ≥ 0.84, and even for the most disparate buffer sizes (200 vs. 1000 m) the correlations all were *r* ≥ 0.7.

**Table 3:** Pearson correlation coefficients (*r*) among 3 different GIS-circular buffer radii for percent agriculture (%Agr), percent developed (%Dev), and percent impervious surface (%Imp) land use/land cover

Buffer radii (m)	%Agr	%Dev	%Imp
200 vs. 500	0.92	0.85	0.84
500 vs. 1000	0.95	0.93	0.93
200 vs. 1000	0.81	0.71	0.70

To determine which buffer scale was most predictive of wetland responses, we focused on %Agr correlations with VMMI, because these variables had one of the stronger correlations in the data and no missing values. Overall, there was little difference in correlation using data collected at the 200-, 500-, or 1000-m buffer sizes (Table 4), although the 1000-m buffer had slightly higher correlation coefficients both nationally (*r* = − 0.43 versus − 0.41 or − 0.38) and for many of the subpopulations. Because these analyses did not find important differences among buffer sizes, just one buffer size was chosen for further analyses, which also avoids potential problems with multicollinearity among LULC predictors. The largest buffer size (1000 m) integrates a larger area and had slightly higher correlations so we only used the largest buffer size data in all subsequent analyses.

**Table 4:** Comparison of Pearson correlations (*r*) between vegetation multimetric index (VMMI) and percent agriculture for three GIS buffer radii

Subpopulation	200-m radius	500-m radius	1000-m radius
National	− 0.38	− 0.41	− 0.43
NWCA aggregated ecoregion			
Coastal Plain	− 0.41	− 0.42	− 0.42
Eastern Mountains and Upper Midwest	− 0.44	− 0.48	− 0.50
Interior Plains	− 0.22	− 0.23	− 0.26
West	− 0.18	− 0.22	− 0.24
NWCA aggregated wetland type			
Estuarine herbaceous	− 0.07	− 0.08	− 0.18
Estuarine woody	− 0.16	− 0.16	− 0.13
Palustrine, riverine, lacustrine-herbaceous	− 0.31	− 0.34	− 0.37
Palustrine, riverine, lacustrine-woody	− 0.40	− 0.40	− 0.39
HGM class			
Depressions	− 0.32	− 0.34	− 0.35
Flats	− 0.45	− 0.52	− 0.54
Riverine	− 0.34	− 0.35	− 0.33

### Patterns and relationships for disturbance and response variables

**Fig. 2:**
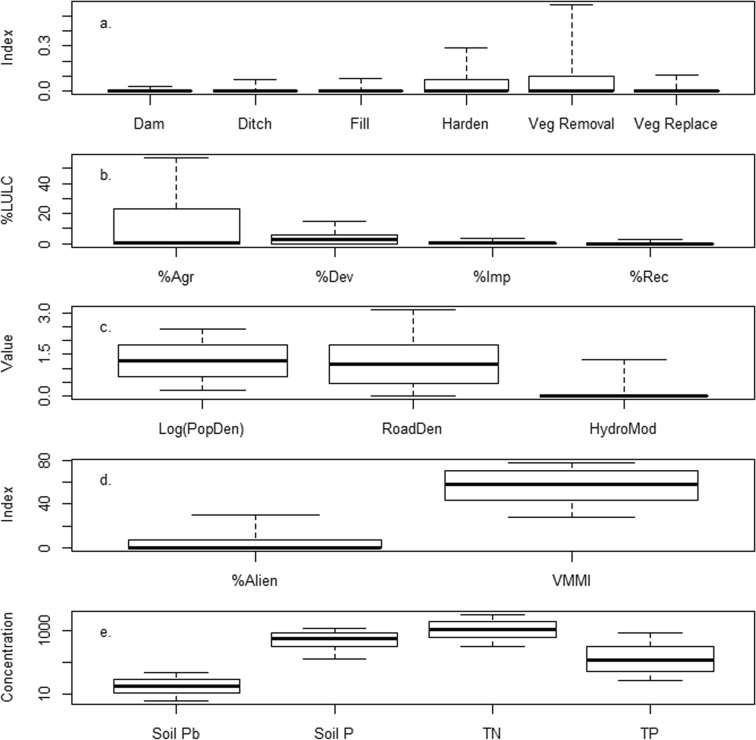
Box and whisker plots showing the distribution of National Wetland Condition Assessment data across all sampled sites for **a.** index values for site-level disturbance variables; **b.** % land use/land cover (%LULC) for GIS landscape disturbance variables in the 1000-m buffer; **c**. GIS landscape disturbance values for log10 population density (number/mi^2^), untransformed road density (km/km^2^), and untransformed hydrologically modified length (km) in the 1000-m buffer; **d.** index values for % alien cover and vegetation multimetric index (VMMI); and **e.** concentrations of soil lead (Pb) and soil phosphorus (P) in mg/kg, and water column total nitrogen (TN) and total phosphorus (TP) in μg/L. Boxes show the median and interquartile range; whiskers show the 10th/90th percentiles. Points beyond the 10th and 90th percentiles are not plotted. Variable codes are given in Table 2

The range of values observed for disturbance and response variables across the NWCA sites sampled in the conterminous US varied widely (Fig. 2), as might be expected for such a large-scale survey. Among the site-level disturbances (Fig. 2a), zero values were quite common (i.e., no disturbance observed in any buffer plot at a site). For the ditching, damming, filling/erosion, and vegetative replacement indices, 78–87% of the sites had zero index values. Vegetative removal and hardening were the most commonly observed site-level disturbances with each present at some level in about 40% of the sites. Nationally, the median index value for all site-level disturbance indices was zero. Among the GIS landscape disturbance variables (Fig. 2b and c), median percent agriculture was 0.11 (interquartile range (IQR) = 0–23.4) and median percent developed land was 2.6 (IQR = 0–5.4). Median population density was 18.4 people/mi^2^ (IQR = 4.8–70.3) and median road density was 1.17 km/km^2^ (IQR = 0.46–1.84). There were many zero values in the landscape data. Over 85% of the sites had no recreation disturbance or hydrologically modified length. Similarly, 49% of the sites had no agricultural LULC, 28% had no development, and 22% had no impervious surface. A much lower percentage of sites had zero population (0.8%) or zero road density (11%). Even when non-zero, percent developed and impervious land cover were quite low (< 10%) in the vast majority of sites (Fig. 2b). VMMI and percent alien cover data ranged widely, with minimum values of zero and maximum values over 90, but the IQR for VMMI was 44–70 versus 0–7 for percent alien cover (Fig. 2d). Median soil concentrations for P (556 mg/g) were higher than those for lead (17.3 mg/kg), whereas for water chemistry, the median concentration of TP was 121 μg/L and TN was 1080 μg/L (Fig. 2e).

The degree of covariance among predictor variables and among response variables, as assessed by using a Pearson correlation of *r* ≥ 0.5 criteria, varied considerably among metrics and among subpopulations. Among the disturbance predictor variables, percent impervious surface, percent developed land, and road density were correlated with each other at *r* ≥ 0.5, both nationally and for all subpopulations. Human population density and road density were correlated at *r* ≥ 0.5 for all subpopulations except HGM flats. The median correlation among all disturbance variables across subpopulations was 0.05, while the maximum correlation (*r* = 0.96) was between percent impervious surface and percent developed land in the IPL. Among the 6 response variables, correlations between TP and TN were always ≥ 0.5. All subpopulations, except the EMU (*r* = − 0.48), had negative correlations ≥ 0.5 between VMMI and percent alien species. Soil lead and P were not correlated with each other or any of the other wetland response variables.

**Fig. 3:**
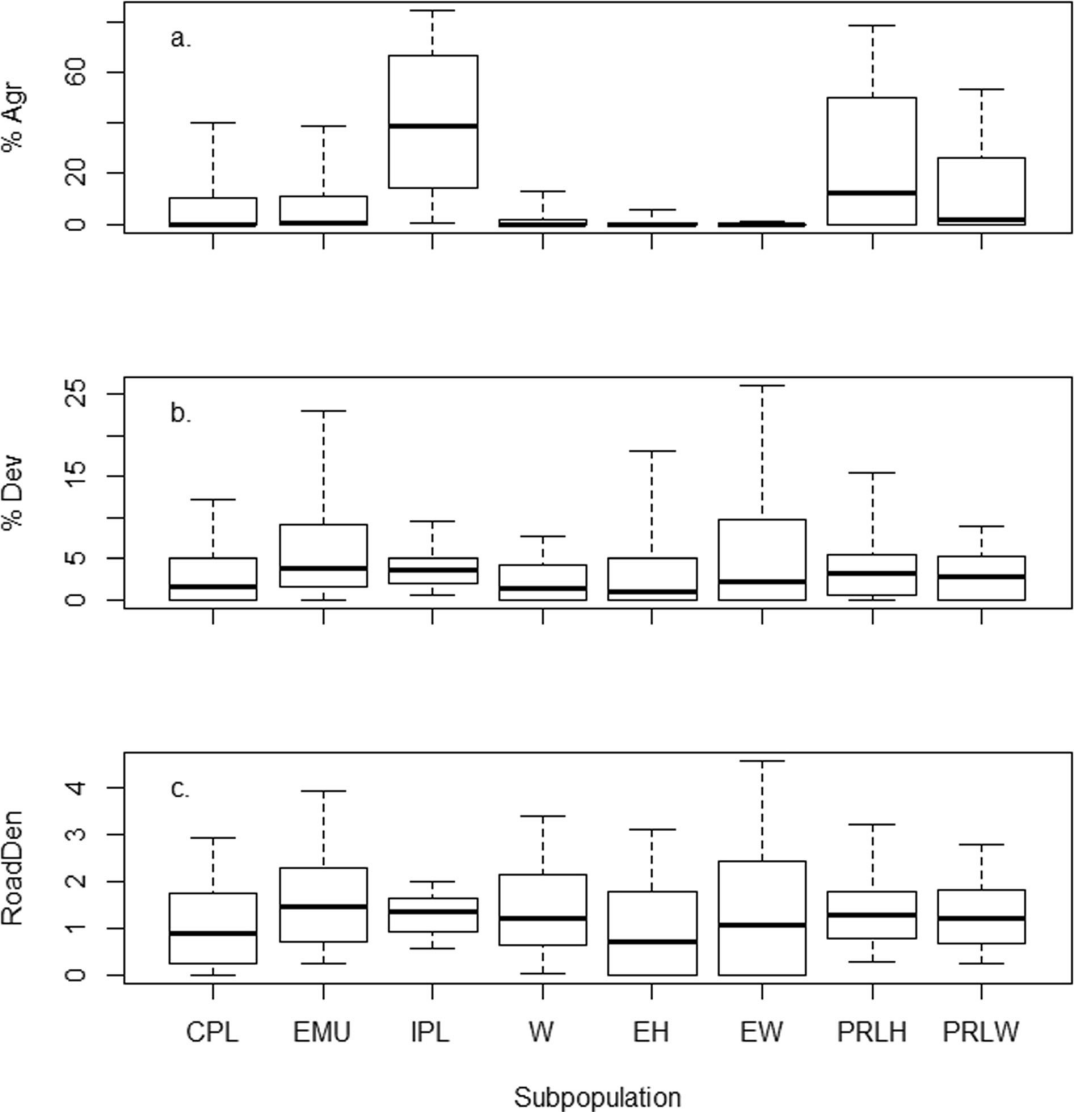
Box and whisker plots showing the distribution of (**a**) percent agricultural land (%Agr), (**b**) percent developed land (%Dev), and (**c**) road density (RoadDen in km/km^2^) in the 1000-m radius buffer for the four NWCA aggregated ecoregions and four aggregated wetland types. See Table 1 for definition of ecoregion and wetland type codes. Boxes show the median and interquartile range; whiskers show the 10th/90th percentiles. Points beyond the 10th and 90th percentiles are not plotted

The distribution of percent agriculture in the 1000-m buffer varied greatly among different wetland subpopulations (Fig. 3a). It was significantly different across ecoregions (one-way ANOVA *F* = 115, *p* < 0.0001), wetland types (*F* = 72.3, *p* < 0.0001), and HGM classes (*F* = 36.2, *p* < 0.0001). Agriculture was virtually absent in the surrounding 1000-m radius area for estuarine EH and EW sites, but common in the inland PRLH vegetation types (Fig. 3a). Among ecoregions, percent agriculture was high in the IPL (≥ 10% for 82% of sites) but rare in the West (≥ 10% for 13% of sites). Sites in the CPL and EMU had similar distributions of percent agriculture, which were intermediate to levels seen in the IPL and W. Variability in percent developed land among wetland subpopulations (Fig. 3b) was much lower than was observed for percent agriculture. Among ecoregions, it was highest in the EMU. Distributions of road density were similar across both ecoregion and wetland type (Fig. 3c).

### Efficacy of site-level versus GIS landscape disturbance variables in regressions

**Fig. 4:**
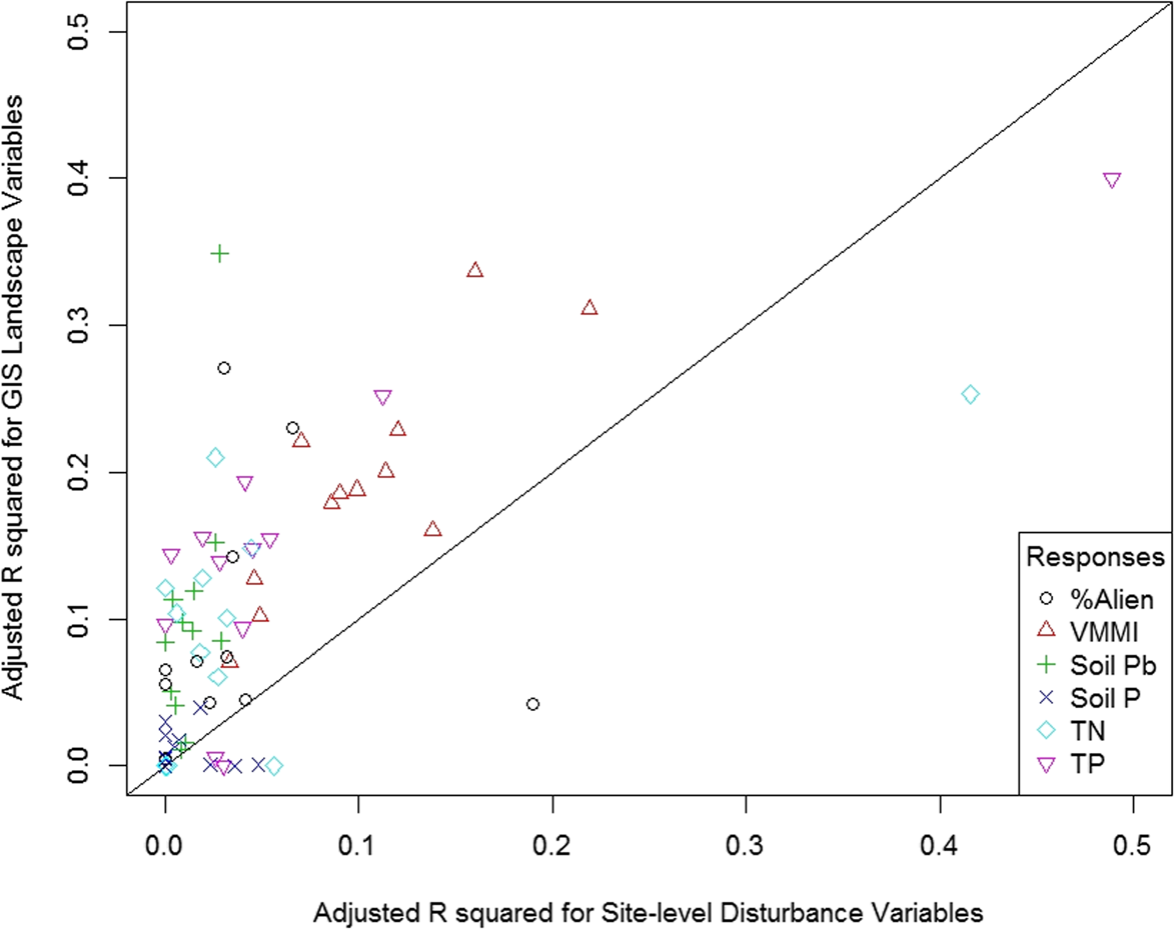
Adjusted *R*-squared values for multiple regression models built using only GIS landscape disturbance variables versus models built using only site-level disturbance variables. Response variable codes are listed in Table 2

Separate multiple-regression models using only site-level or only GIS landscape-level disturbance variables suggested that landscape-level predictors had somewhat higher explanatory power (Fig. 4). The adjusted *R*^2^ values for the models with GIS landscape predictors only were higher than those for models with site-level predictors for 62 of the 72 model comparisons run (1 national and 11 subpopulation models for each of 6 predictor variables). Typically, landscape-only models had an adjusted *R*^2^ between 0.01 to 0.1 units higher than site-level-only models. For response variable and subpopulation combinations where 10% or more of the overall variance was explained, the *R*^2^ for GIS landscape-level predictors typically exceeded those of site-level predictors by 10–20 percentage points, and only three site-level-only models had a higher adjusted *R*^2^ than landscape-only models (Fig. 4).

Among the 72 separate site-level and GIS landscape models, 12 were considered significant having adjusted *R*^2^ ≥ 0.2 and *p* values ≤ 0.0003; nine of these fit the data reasonably well as determined by visual inspection of the residual plots. The residuals were randomly dispersed about the zero line and we did not see evidence of non-constant variance, non-linearity, or large outliers in the plots. Among these nine models, only one, the response-subpopulation combination of the VMMI in the EMU, had significant models for both the landscape-only and site-level-only predictors. For this model, the extra sum of squares *F*-test for the site-level variables (*p* value < 0.00000001) showed there was a strong difference between the full and reduced models, and that the full model (i.e., with both field and GIS variables) was better. Similarly, the extra sum of squares *F*-test for the GIS landscape variables alone compared to the full dataset (*p* value = 0.0004) also shows the full model performing better. Consequently, we used the full models, including site-level and landscape disturbance variables, for the remaining analyses.

### Disturbance-response multiple regression models

**Fig. 5:**
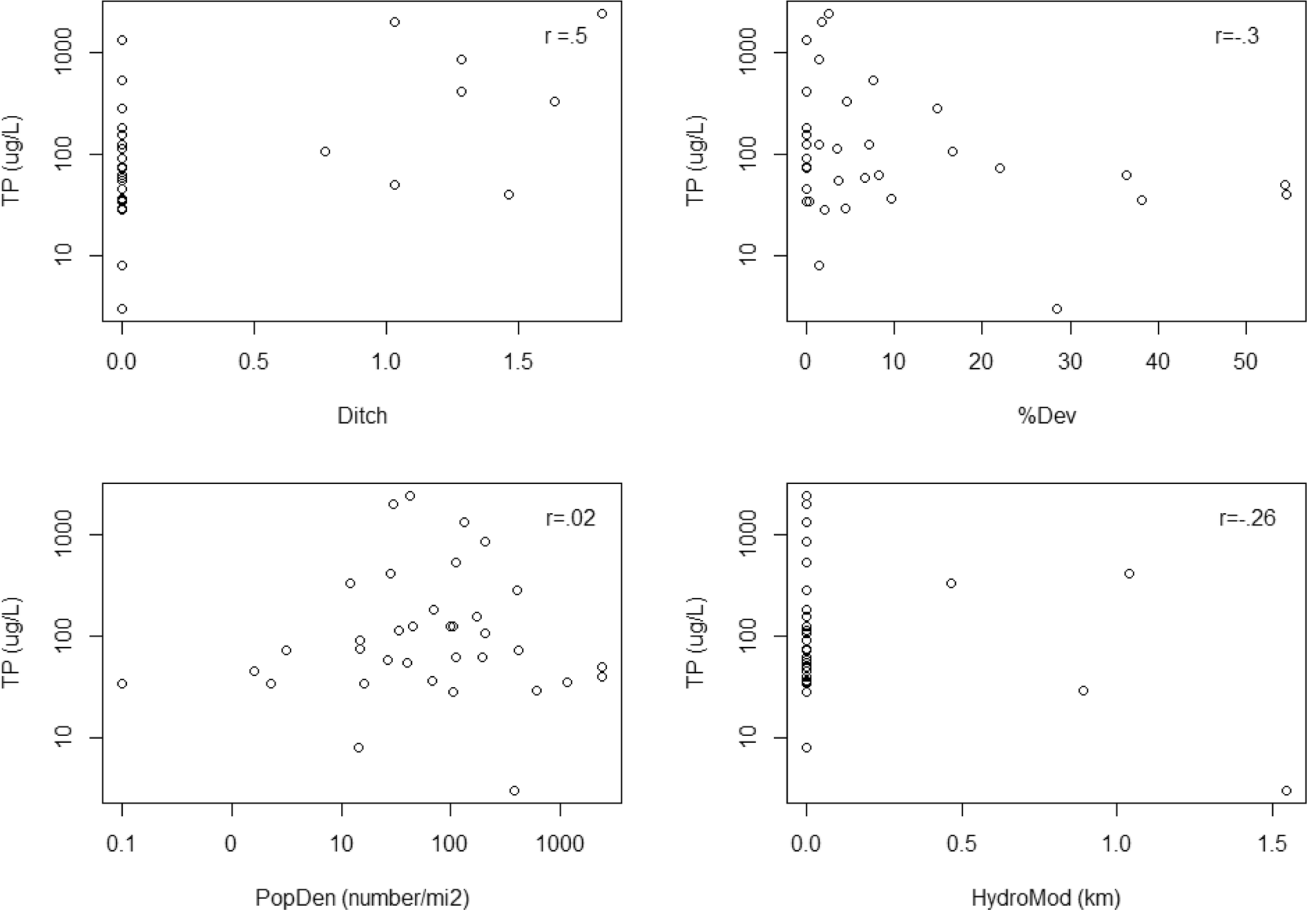
Scatter plots showing the relationship between water column total phosphorus (TP) and the disturbance variables in the estuarine-woody wetland type. Disturbance variable codes are given in Table 2

**Fig. 6:**
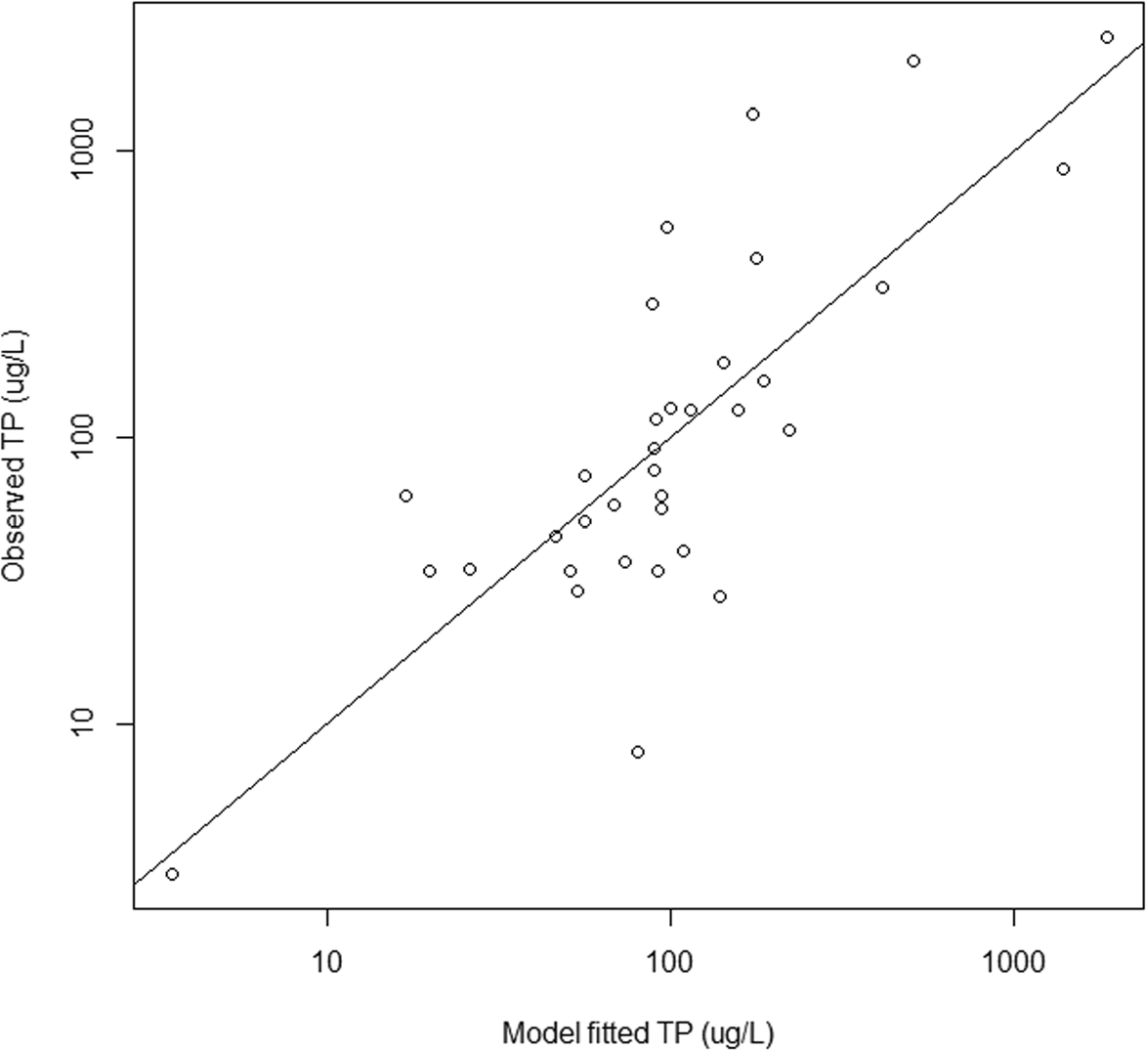
Observed water column total phosphorus (TP) versus multiple regression model predicted TP in the estuarine-woody wetland type

For the final multiple regression analyses, we included both site-level and GIS landscape disturbance variables and built a single model for each of the six response variables at the national scale and for each of the subpopulations. Out of the 72 resulting models, 12 passed the significance screening criteria (adjusted *R*^2^ ≥ 0.2 and *p* values ≤ 0.0003, Table 5). The response variable for seven of these models was VMMI. In the other five significant models, the response variable was soil lead once, TP twice, and TN twice. We were not able to build a significant model for soil P or percent aliens. The disturbance predictor variable most commonly observed in the significant models was percent agriculture, found in nine of the 12 models; the next most common predictor was ditching, which occurred in seven models. Significant models were found in all the wetland subpopulations except for EH and CPL. All of the disturbance variables except hardening appeared in at least one of the 12 models. The models had between one and five predictor variables with an average of about four. The model with the highest adjusted *R*^2^ of 0.577 was for TP in the EW subpopulation. For this model, the significant disturbance variables were ditching, percent developed, human population density, and hydrologic modified length. Bivariate scatter plots for these variables versus TP are shown in Fig. 5. Bivariate correlations for these four scatterplots ranged from − 0.3 to + 0.5 with ditching being the strongest correlation. Note that some of these relationships are defined by just a few influential observations. The overall regression model fit (observed versus predicted plot) for this model (TP in EW) is shown in Fig. 6 to illustrate the highest *R*^2^ model fit.

**Table 5:** Significant disturbance-response multiple regression models for subpopulations in the NWCA. Numbers are the regression coefficients for the disturbance variables in each model (variable codes are given in Table 2)

Response Subpopulation	Adj. *R*^2^	Intercept	Dam	Ditch	Fill	VegRemoval	VegReplace	%Agr	%Dev	%Imp	%Rec	PopDen	RoadDen	HydroMod
VMMI														
National	0.251	64.05	− 2.81	− 2.60		− 0.973		− 2.90	− 2.44					
EMU	0.374	65.11					− 6.23	− 3.78	− 2.75					
W	0.260	62.59		− 3.19	− 1.76			− 1.65				− 3.56	− 2.85	
PRLH	0.235	59.41		− 4.15				− 2.45	− 4.00					
PRLW	0.248	62.13		− 3.19		− 1.13		− 2.02					− 2.07	− 1.85
Flats	0.397	64.64		− 4.76	− 1.51			− 3.23		− 8.61				1.83
Riverine	0.229	59.93				− 0.625	− 2.13	− 1.31					− 2.52	− 3.85
TN														
EW	0.409	2.99	0.410	0.285						− 0.221				
PRLW	0.201	2.64						0.052				0.221	− 0.066	
TP														
EW	0.577	1.6		0.688					− 0.289			0.298		− 0.647
Depression	0.246	2.36						0.071		0.313	− 0.072	−0.241		
Soil Pb														
IPL	0.363	1.06										0.169

### Disturbance-response logistic regression models

We were able to build significant logistic regression models (*p* < 0.0003) to predict the presence/absence of alien species at a site at the national scale and for 10 of the 11 wetland subpopulations (Table 6). The IPL was the only subpopulation lacking a significant logistic regression relationship for alien species. Models had between one and six variables with an average of three variables per model. Hardening and percent agriculture were highly significant variables in the national model and most subpopulation models. McFadden’s *R*^2^ varied between 0.07 (in EH and CPL) and 0.23 (in EMU). Values between about 0.2 and 0.4 suggest a very good fit (McFadden 1978).

**Table 6:** Significant logistic regression models predicting presence/absence of alien species. Numbers are the regression coefficients (odds ratios) for the significant disturbance variables in each model (variable codes are given in Table 2). *R*^2^ is McFaddens *R*^2^ for logistic regression

Subpopulation	*R*^2^	Fill	Harden	VegRemoval	VegReplace	%Agr	%Dev	%Imp	PopDen	RoadDen
National	0.15	1.30	1.29*	1.13		1.29*			0.73*	1.30*
CPL	0.07		1.33*			1.21*				1.16
EMU	0.23	2.55			3.05	2.07*				
W	0.10		1.66							
EH	0.07	1.37				3.17*				
EW	0.20	2.78						0.20	5.08	
PRLH	0.12			1.25*		1.23*		3.27		
PRLW	0.11		1.46*			1.22*	0.62*			1.75*
Depression	0.11	2.06				1.17			0.53	1.61*
Flats	0.14		1.32			1.43*				
Riverine	0.08		1.43	1.23						

*Variable significant in model at *p* < 0.001, all other variables significant at *p* < 0.05

The logistic regression coefficients in Table 6 can be interpreted as odds ratios. For every one unit increase in the value of the disturbance variable, it is estimated that the odds of having alien species present as opposed to absent is *X* times more likely (where *X* is the odds ratio). Recall that we transformed the disturbance variables for regression analysis so that a unit change in the percentage variables is 10 percentage points (percent divided by 10) and a unit change in population density is a factor of 10 (log10 transformed). These transformations are especially useful for the logistic regression, making the odds ratios more comparable among disturbance variables. The EMU ecoregion had the highest McFadden’s *R*^2^ (0.23) and relatively large values for the odds ratios (Table 6). For every 10-percentage-point increase in percent agriculture in the EMU, it is estimated that it is 2.07 times more likely that a site will have alien plant species. Similarly, for a one-point increase in the vegetative replacement index, it is estimated to be 3.05 times more likely to have a site with alien plant species present.

It was also possible to build significant logistic regression models, at the national scale and for nine subpopulations, to predict surface concentrations of lead in wetland soils that were above or below the NWCA threshold background concentration of 35 mg/kg (Table 7). The only subpopulations without a significant model were the EW wetland type and the flats HGM class. The logistic regression models had between one and four predictor variables with an average of two per model, and McFadden’s *R*^2^ ranged between a low of 0.07 in the CPL and PRLW and a high of 0.34 in the IPL. Human population density was a highly significant variable in all the individual logistic regression models except in the W ecoregion and in the EH wetland type where hydrologic modification and percent developed land were significant variables. Nationally, for a 10-fold increase in population density, it is estimated that it would be 2.31 times more likely to have lead in wetland soils above background (Table 7). The odds ratios for population density in the individual subpopulation models ranged from 2.11 (PRLW) to 7.40 (IPL).

**Table 7:** Significant logistic regression models predicting wetland soil lead concentration above/below a background concentration of 35 mg/kg. Numbers are the regression coefficients (odds ratios) for the significant disturbance variables in each model (subpopulation codes are given in Table 1, variable codes are given in Table 2). *R*^2^ is McFaddens *R*^2^ for logistic regression

Subpopulation	*R*^2^	Fill	VegRemoval	%Agr	%Dev	%Imp	%Rec	PopDen	HydroMod
National	0.1			0.87			1.12	2.31*	
CPL	0.07							2.67*	
EMU	0.11		0.73					2.29*	
IPL	0.34							7.40*	
W	0.11								1.26*
EH	0.16				2.22*				1.34
PRLH	0.19			0.81		0.44		4.34*	
PRLW	0.07			0.83				2.11*	
Depressions	0.21	0.52		0.79		0.41		4.85*	
Riverine	0.08							2.73*	

*Variable significant in model at *p* < 0.001, all other variables significant at *p* < 0.05

## Discussion

### Effect of GIS buffer radius on landscape-level disturbance

The high correlation among land-cover composition within 200-, 500-, and 1000-m circular buffers around each site makes it difficult to establish any one buffer size as being the best predictor of wetland disturbance (Table 3). This makes it very difficult to determine patterns regarding which land-cover buffer size was most related to disturbance. Pearson correlation coefficients between percent agricultural land and stressors were all within a few hundredths of each other for the different buffer sizes (Table 4). Thus, at the national scale surveyed by the NWCA, it does not appear that any one of the tested buffer sizes performs significantly better than another, and we built all of our models using the 1000-m landscape buffer data as it had slightly higher correlations and integrated a larger surface area. Brazner et al. (2007) studied the responsiveness of Great Lakes wetland indicators at multiple spatial scales and found that most biological assemblages responded to disturbances characterized at larger spatial scales (1000 m and whole-watershed scale), and that the 100-m buffer scale was relatively uninfluential. Since the 1000-m scale is the largest we tested here, we cannot rule out the possibility that still larger spatial scales would improve predictions even for the VMMI. It would have been interesting to test watershed scale variables with our data but it was not logistically feasible to delineate watersheds for all 1138 sites. On the other hand, Rooney and Bayley (2011) and Rooney et al. (2012) reported that small buffers and local conditions were better predictors of plant diversity and plant IBI scores in Alberta wetlands than were larger buffers and landscape condition, and Galatowitsch et al. (2000) found local disturbance more influential to plant composition in Minnesota prairie wetlands than distal disturbance. Studies that have compared multiple wetland taxonomic groups (e.g., plants, amphibians, macroinvertebrates) have found that different organisms respond to different aspects of disturbance at different scales (e.g., Mensing et al. 1998; Findlay and Houlahan 1997; Brazner et al. 2007) which must be taken into account for effective conservation planning.

### Disturbance-response relationships

The generally low percentage of models having significant *R*^2^ and the wide variation in predictor variables selected suggests that stressor-response relationships vary considerably across the diversity of wetland types and landscape settings found across the conterminous US. Of the 72 possible models, 12 were significant when adjusted for multiple comparisons and only five explained over 30% of the variance (adjusted *R*^2^ > 0.3) (Table 5). The composition of the significant predictor disturbance variables incorporated into each model varied widely by subpopulation and response variable. The relatively low *R*^2^ values for significant relationships are not surprising, when considering the large scale of the NWCA. The wide spatial variance and range of different natural and anthropogenic factors that can affect the response variables likely make it impossible to achieve high *R*^2^ values with these kinds of regression models. In addition, some of the wetland subpopulations, in particular the estuarine ones, had a high proportion of comparatively low-disturbance sites (based on the NWCA disturbance gradient) leaving little signal to model (Herlihy et al. 2019). We are not aware of any other wetland analyses at the spatial scale of the NWCA, but in similar large-scale lake and stream survey analyses, water chemistry-disturbance relationships rarely had *R*^2^ values over 0.4 and were more typically in the 0.2–0.3 range (Herlihy et al. 1998, Herlihy et al. 2013; Herlihy and Sifneos 2008; ). With large comparative datasets as the NWCA, *R*^2^ values < 0.1 can be statistically significant due to large sample sizes, leaving open the matter of their ecological significance.

Despite the large-scale and the accompanying variability of the NWCA, we were able to build significant stressor response models for the VMMI, nationally and for six of the 11 wetland subpopulations. Significant subpopulation models included two ecoregions (EMU, *R*^2^ = 0.374, and W, *R*^2^ = 0.260), two NWCA aggregated wetland types (PRLH, *R*^2^ = 0.235, and PRLW, *R*^2^ = 0.248), and two HGM classes (flats, *R*^2^ = 0.397, and riverine, *R*^2^ = 0.229) (Table 5). All the disturbance variables we assessed were significant in one of the VMMI regression models except for hardening. Percent agriculture and ditching were the variables present in the majority of VMMI models. Adjacent agriculture would likely provide propagule sources to wetlands, and both agriculture and ditching could provide dispersal vectors into wetlands; these two factors (propagule abundance and dispersal routes) have been identified as the two main ways in which adjacent land use affects wetland plant communities (Houlahan et al. 2006). Note that the VMMI was developed using NWCA least- and most-disturbed sites to select and score individual vegetation metrics (Magee et al. 2019a) so it may be more tuned to the field-based stressor gradient than the other response indicators. Least- and most-disturbed NWCA sites were defined, in part, using indices calculated from the NWCA buffer and heavy-metal data but not GIS landscape measures or water chemistry (Herlihy et al. 2019).

Other researchers have found stronger correlations between wetland vegetation condition and disturbance in smaller scale surveys. In Ohio, Stapanian et al. (2013) analyzed 20 disturbance variables from 149 wetlands to predict a vegetation index of biotic integrity (IBI). They found a model *R*^2^ of 0.61 for emergent wetlands, 0.54 for forested wetlands, but no significant model for shrub wetlands. They reported that the IBI was better predicted by wetland-scale measures, specifically substrate and habitat disturbance, as opposed to measures of the surrounding landscape. However, Mack (2006) reported regression models of similar strength as Stapanian et al. (2013) for the same three Ohio wetland classes relating the vegetation IBI to a landscape disturbance index based only on remote sensing data. In Florida, a floristic quality assessment index (FQAI) for 75 depressional herbaceous wetland systems was related to adjacent 100-m buffer land-use intensity by Cohen et al. (2004). They reported an *R*^2^ of 0.48 with similar results across northern, central, and southern Florida ecoregions. Plant richness was correlated with road density and forest cover using multiple regression in Southeastern Ontario wetlands with similar *R*^2^ results (0.56–0.63) across a range of buffer sizes from 250 to 2000 m (Findlay and Houlahan 1997). We think the difference in correlation strength observed in the NWCA compared to these smaller scale surveys has to do with the degree of heterogeneity in wetland types and landscape setting. In a larger scale survey of Great Lakes wetlands, Brazner et al. 2007 found relationships between wetland vegetation and disturbance variables of similar strength to those we observed in the NWCA. They found that percent row crop agriculture and development were the most important predictors.

We did not find significant national models for percent aliens, soil P, soil lead, TP, or TN, and neither percent aliens nor soil P concentration had significant models for any of the subpopulations. However, significant models for TP, TN, and soil lead were obtained for a few individual wetland subpopulations. Soil and water chemistry may be more strongly related to factors outside the scope of the site-level and landscape variables assessed in the NWCA. For example, in a more detailed analysis of the NWCA water chemistry data, Trebitz et al. (2019) found the strongest relationships between water quality and land use at the basin level (12-digit hydrologic unit code), rather than the 200–1000-m buffers used for NWCA GIS data. Comparative studies of water chemistry across wetlands of the Laurentian Great Lakes have likewise found water quality responsive to LULC across large spatial scales (Crosbie and Chow-Fraser 1999; Trebitz et al. 2007). Houlahan and Findlay (2004) also concluded that effects of land use on wetland sediment and water quality can extend over comparatively large distances, and that effective conservation would not be achieved with the creation of narrow buffer zones alone. It is also likely that responses to disturbance for soil and water chemistry are more complex than simple linear relationships with land-cover composition or the NWCA indices describing site-level disturbance intensity. Trebitz et al. (2007) utilized an index of agricultural intensity based on principal components analysis that had stronger relations to wetland nutrient concentrations than those reported by others in the same region using simple percent agriculture as a disturbance measure.

Based on results of the multiple regression models, it appears that wetland vegetation condition was more strongly related to both the site-level and landscape-level disturbances measured in the NWCA than were the other five response variables. It should be emphasized that our aim in these multiple regression analyses was to look for large-scale associations between wetland disturbance and response variables, rather than to build predictive models of the response variables. A thorough predictive model would likely require the addition of natural driver variables (e.g., hydroperiod, temperature, elevation) to the model to improve model performance by accounting for underlying natural gradients and separating them from disturbance effects (Brazner et al. (2007)).

We chose multiple regression for evaluating disturbance-response relationships because it yields results that are easy to compare across response variables and wetland subpopulations. In our analyses, we address the influence of natural factors by using different wetland subpopulations to minimize within-group variance (Herlihy et al. 2019). It is also important to note that our results focus only on one set of predictor variables that might be used to describe the response variables. Our analysis does not imply that these disturbances are causal variables nor that they are necessarily the “best” set of predictor variables.

### Site-level versus GIS landscape disturbance variables

The best multiple regression models included both site-level and landscape disturbance variables. However, we were also interested in examining whether regression models based solely on site-level field data performed differently than models based solely on GIS landscape variables (Fig. 4). Across all six response variables and all subpopulations examined, landscape-only models and site-level-only models often explained little of the variance (*R*^2^ < 0.2). In the five cases where either type of model had an *R*^2^ over 0.3, there was no clear pattern of which performed better (landscape performed better three times, site-level twice). Overall, landscape-only models tended to have about 0.05–0.1 higher *R*^2^ than site-level-only models but that was typically a difference of ~ 0.15 versus 0.05. Our analyses suggest that both scales of disturbance data are important in predicting wetland responses.

### Predicting alien plant presence and soil lead in wetlands

We were able to build significant national and subpopulation logistic regression models predicting site presence/absence of alien species and soil lead above/below the NWCA background concentration of 35 mg/kg, despite the fact that we could not build a significant national linear regression model for either of them as continuous variables. It may be that anthropogenic disturbance predicts whether alien species or lead gets into the wetland to begin with but that there is a very different set of factors that explain the actual percentage cover of alien species or soil lead concentrations. For example, local biogeochemical processing and hydrology may be what explains lead concentration once it is actually present in the wetland. Similarly, the actual percentage of alien cover may be driven primarily by within-wetland factors such as resistance to their establishment by other plants and not disturbance variables.

Landscape percent agriculture was the most common variable in the logistic regression models predicting the presence/absence of alien plant species and is the most universal indicator, among our study variables, of the potential for alien species invasions. The site-level disturbance indices for filling/erosion and hardening were also significant predictors. All three of these variables were in the national model and one, two, or all three of them were retained as predictors in models across the various wetland subpopulations. All of the tested disturbance variables strongly reflect human influence and are possible pathways for alien species to get into the study wetlands, filling with soil from outside the wetland potentially introduces seed sources, and hardening disturbs the native plant community and potentially allows adventive species a competitive advantage (Hobbs and Huenneke 1992; McIntyre and Lavorel 1994).

For predicting the occurrence of wetlands with surface soil lead above background concentrations, human population density in the 1000-m landscape buffer around the study wetland was the most significant variable, with the highest logistic regression odds ratios. This was somewhat surprising in that we initially thought that road density might be a stronger predictor due to the legacy effect of leaded gasoline. The GIS road layer, however, does not discriminate among road types (e.g., freeway versus back road), so perhaps population density is a better proxy for traffic volume than road density. Road density did not appear in any of the lead models. While several other disturbance variables were retained in one or more predictive models, none appeared as frequently as population density and their odds ratios were not nearly as high.

## Summary and conclusions

The landscape disturbance indicators were highly correlated among the 200-, 500-, and 1000-m radius circular landscape buffers and gave similar results when related to response indictors, but the larger 1000-m buffer generally gave slightly stronger predictions. Thus, only the 1000-m buffer data were used for subsequent analyses. Disturbance-response models built using only landscape variables or only site-level variables often explained only a small portion of the variance in the response variable (*R*^2^ < 0.2). Overall, landscape-only models tended to have about 0.05–0.1 higher *R*^2^ than site-level-only models. Our analyses suggest that both types of disturbance data are important in predicting wetland responses as the strongest regression models contained both site-level and landscape disturbance variables. The VMMI was the response variable that was most related to the disturbances we assessed (national model *R*^2^ = 0.251). Percent agriculture and ditching were the disturbance variables that occurred in most of the VMMI models across the different wetland subpopulations. National multiple linear regression models for the soil and water chemistry, and percent alien cover responses, were not significant, but it was possible to build significant models predicting presence/absence of alien species and presence of soil lead above/below the NWCA background concentration of 35 mg/kg in many of the tested wetland subpopulations. This suggests that disturbance determines whether alien species and lead are present but that processes other than disturbance (e.g., wetland species composition, biogeochemistry) control their abundance or concentration once present.
